# Supplementary figures and images for: Overweight and obesity association with mortality in patients with heart failure and reduced or preserved ejection fraction-a cohort study
Source: PLoS One. 2026 Feb 3;21(2):e0341606. doi: 10.1371/journal.pone.0341606 (PMC12867239; doi:10.1371/journal.pone.0341606)

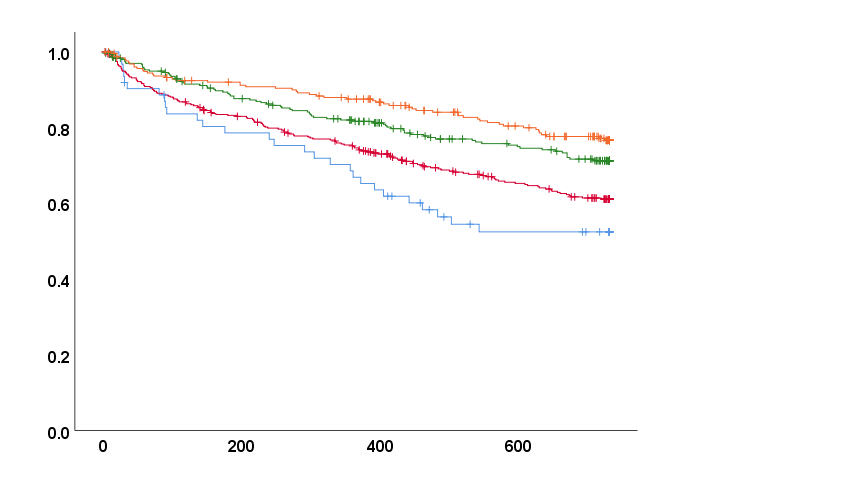

Supplement: S1 Fig — (TIF) [file pone.0341606.s001.tif]

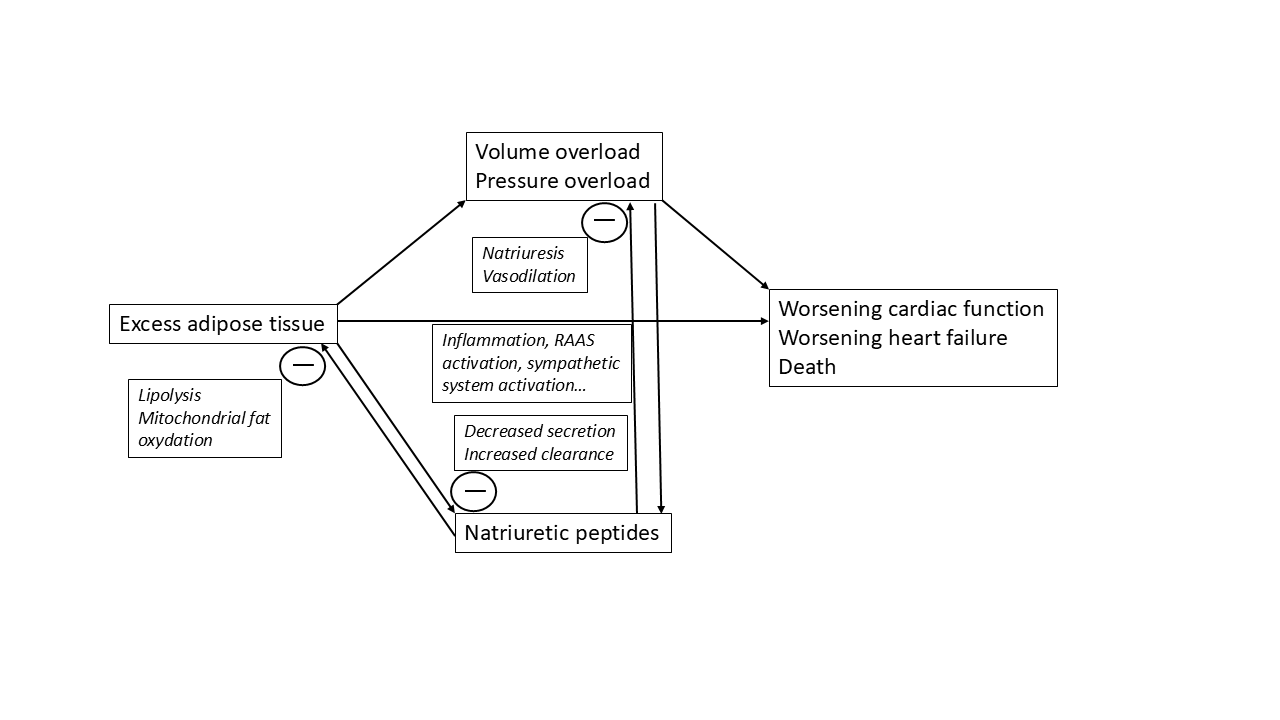

Supplement: S2 Fig — (TIF) [file pone.0341606.s002.tif]
